# Supplementary material for: Prevalence and risk factors for falls among community-dwelling adults in Riyadh area
Source: PeerJ. 2023 Dec 6;11:e16478. doi: 10.7717/peerj.16478 (PMC10710170; doi:10.7717/peerj.16478)
Supplement: Supplemental Information 3 [file peerj-11-16478-s003.docx]

**Code Book for the Prevalence and risk factors for falls among community-dwelling adults aged 40 years or older in Saudi Arabia^[[1]](#footnote-1)^**

Marital Status:

Single 1

Married 2

Divorced 3

Widowed 4

**Sex:**

Female 0

Male 1

**Education:**

None 0

Elementary 1

Middle 2

Secondary 3

University 4

**Occupation:**

Unemployed 0

Employed 1

Retired 2

**Smoking:**

Yes 1

No 0

**Chronic conditions all:**

Yes 1

No 0

**Fall history:**

Yes 1

No 0

**Number of falls:**

Add numbers such as 0, 1, 2, 3,…etc

**BdItotal (Beck Depression Inventory)**

Add the score

1. [↑](#footnote-ref-1)
